# Distinct Physiological Roles of the Three Ferredoxins Encoded in the Hyperthermophilic Archaeon *Thermococcus kodakarensis*

**DOI:** 10.1128/mBio.02807-18

**Published:** 2019-03-05

**Authors:** Brett W. Burkhart, Hallie P. Febvre, Thomas J. Santangelo

**Affiliations:** a Department of Biochemistry and Molecular Biology, Colorado State University, Fort Collins, Colorado, USA; University of California, Irvine

**Keywords:** *Thermococcus kodakarensis*, archaea, electron flux, ferredoxin, hydrogenase, hyperthermophile

## Abstract

Control of electron flux is critical in both natural and bioengineered systems to maximize energy gains. Both small molecules and proteins shuttle high-energy, low-potential electrons liberated during catabolism through diverse metabolic landscapes. Ferredoxin (Fd) proteins—an abundant class of Fe-S-containing small proteins—are essential in many species for energy conservation and ATP production strategies. It remains difficult to model electron flow through complicated metabolisms and in systems in which multiple Fd proteins are present. The overlap of activity and/or limitations of electron flux through each Fd can limit physiology and metabolic engineering strategies. Here we establish the interplay, reactivity, and physiological role(s) of the three ferredoxin proteins in the model hyperthermophile Thermococcus kodakarensis. We demonstrate that the three loci encoding known Fds are subject to distinct regulatory mechanisms and that specific Fds are utilized to shuttle electrons to separate respiratory and energy production complexes during different physiological states. The results obtained argue that unique physiological roles have been established for each Fd and that continued use of T. kodakarensis and related hydrogen-evolving species as bioengineering platforms must account for the distinct Fd partnerships that limit flux to desired electron acceptors. Extrapolating our results more broadly, the retention of multiple Fd isoforms in most species argues that specialized Fd partnerships are likely to influence electron flux throughout biology.

## Abstract (precis)

High-energy electrons liberated during catabolic processes can be exploited for energy-conserving mechanisms. Maximal energy gains demand these valuable electrons be accurately shuttled from electron donor to appropriate electron acceptor. Proteinaceous electron carriers such as ferredoxins offer opportunities to exploit specific ferredoxin partnerships to ensure that electron flux to critical physiological pathways is aligned with maximal energy gains. Most species encode many ferredoxin isoforms, but very little is known about the role of individual ferredoxins in most systems. Our results detail that ferredoxin isoforms make largely unique and distinct protein interactions *in vivo* and that flux through one ferredoxin often cannot be recovered by flux through a different ferredoxin isoform. The results obtained more broadly suggest that ferredoxin isoforms throughout biological life have evolved not as generic electron shuttles, but rather serve as selective couriers of valuable low-potential electrons from select electron donors to desirable electron acceptors.

## INTRODUCTION

The energy production strategies supporting growth of hyperthermophilic archaea push the known limits of energy-conserving mechanisms (1–7). Although fermentation of peptides and sugars permits net ATP production, many hyperthermophiles are reliant on additional ATP production through the action of respiratory complexes for rapid and efficient growth (8–15). If reduction of even weakly energetic substrates can be coupled to formation of an electrochemical gradient, this gradient can be exploited for ATP production (16–18). High-energy electrons, liberated during amino acid or sugar fermentation, can be used to generate electrochemical ion gradients when delivered to terminal electron acceptors at membrane-bound respiratory complexes. The availability of terminal electron acceptors often changes in natural environments, and thus, many microbes encode alternative routes for electron disposal, each linked to different membrane-bound complexes that reduce the terminal electron acceptor and generate an electrochemical gradient (1, 2, 9).

Catabolic liberation of high-energy electrons requires that these electrons are captured—albeit temporarily—by suitable small molecule or proteinaceous electron carriers. NAD (NAD^+^; NADH when reduced) and NADP (NADP^+^; NADPH when reduced) are common electron carriers, but the utility of NAD(P)^+^ is limited in many scenarios given its thermo-instability (19). In addition, the midpoint electrical potentials of just ∼−320 mV limit the reducing power of NAD(P)H. As such, reduction of protons directly from NADH is not energetically possible; the midpoint potential of H_2_/H^+^ is −414 mV (5).

Given the limitations of NAD(P)^+^, many species encode proteinaceous electron carriers, the most common and abundant of which are termed ferredoxins (Fds) (20–23). Fds, along with related proteins such as flavodoxins, rubredoxins, thioredoxins, and glutaredoxins, provide stable redox electron carriers in hyperthermophilic environments (24). These typically small (>∼20-kDa) proteins coordinate Fe-S clusters that can ferry electrons through redox reactions *in vivo*. Fds provide a wide range of utility, with midpoint electric potentials ranging from nearly equal to those of NAD(P)^+^ at ∼−320 mV to ∼−700 mV under exceptional circumstances (6, 20, 25, 26). Importantly, many Fds are capable of reducing protons to hydrogen, and the availability of protons as a terminal electron acceptor is critical for many biological systems.

In most instances, electron flow through dinucleotide or proteinaceous carriers is regulated to maximize energy gains dependent on environmental conditions and the availability of different electron acceptors. Physiological changes and metabolic shifts are necessarily constrained by the requirement for NAD(P)H for many reactions. The near-ubiquitous use of NAD(P)^+^ for many cellular redox reactions means that general metabolism can be shifted by expression or elimination of a single NAD(P)H utilization complex, but that electron flow cannot be easily directed to a low-abundance complex when necessary without concomitant elimination of high-abundance NAD(P)H-utilizing complexes. The use of multiple Fds, as is common in most microbes and even higher eukaryotes (20), especially higher plants (21, 22, 26–28), theoretically permits electron flux to be tailored between donors and acceptors by specialized Fds that uniquely interact with different redox enzymes.

Our understanding of Fds is complicated by difficulties in defining Fds from primary sequences (20), the demonstrated utility of some Fds to interact with and donate electrons to nonnative redox enzymes with high efficiency (29–32), and the limited range of experimental evidence in support of unique biological functions of distinct Fd isoforms (20, 22, 26, 27). Fds encoded in some plants have been recombinantly expressed and evaluated (21, 22), but direct manipulation of Fd activity *in vivo* has received much less experimental attention. Here we exploit the genetic accessibility (33–35) of the marine hyperthermophilic model archaeon Thermococcus kodakarensis to examine the roles of the three encoded Fds under different growth conditions.

The central metabolism of T. kodakarensis utilizes a modified Embden-Meyerhof (EM) pathway wherein glycolysis results in modest net gains in ATP production (1, 8, 10, 36–39). Cellular growth is dependent on membrane-bound respiratory complexes that use reduced Fds (Fd_red_) to generate electrochemical ion gradients that are exploited for additional ATP production (5, 9, 10, 14, 15, 40, 41). Fd_red_ not only act as temporary carriers of valuable electrons to membrane-bound complexes that couple the exergonic transfer of electrons to the simultaneous translocation of ions across the cellular membrane, but also shuttle electrons to soluble ferredoxin:NAD(P)H oxidoreductases that generate NAD(P)H (42) or reductases involved in isoprenoid-based lipid production (43). Given multiple routes of electron disposal and known differences in the expression of Fds and redox donors and acceptors due to the availability of S^0^ (9, 44–47), we anticipated that each Fd may participate in unique redox pathways and thereby establish parallel but nonoverlapping electron disposal routes.

We present evidence that supports this conjecture and establish the interplay, reactivity, and physiological role(s) of the three genetically encoded ferredoxin proteins in T. kodakarensis. We demonstrate that the three loci encoding known Fds in T. kodakarensis (TK1087, TK1694, and TK2012, encoding Fd-2, -1, and -3, respectively) are subject to distinct regulatory mechanisms and that specific Fds are utilized to shuttle electrons to separate respiratory and energy production complexes during different physiological states. Results obtained from *in vivo* protein-protein interactions reveal that each Fd coordinates a unique route for electron disposal and energy production. We extrapolate our findings to suggest that specialized Fds have evolved—in many species—to link dedicated routes of electron flux and permit regulated dissemination of electrons under varied environmental and cellular conditions.

## RESULTS

### *T. kodakarensis* Fds are differentially expressed and harbor unique structures and electrostatic surfaces.

**FIG 1:**
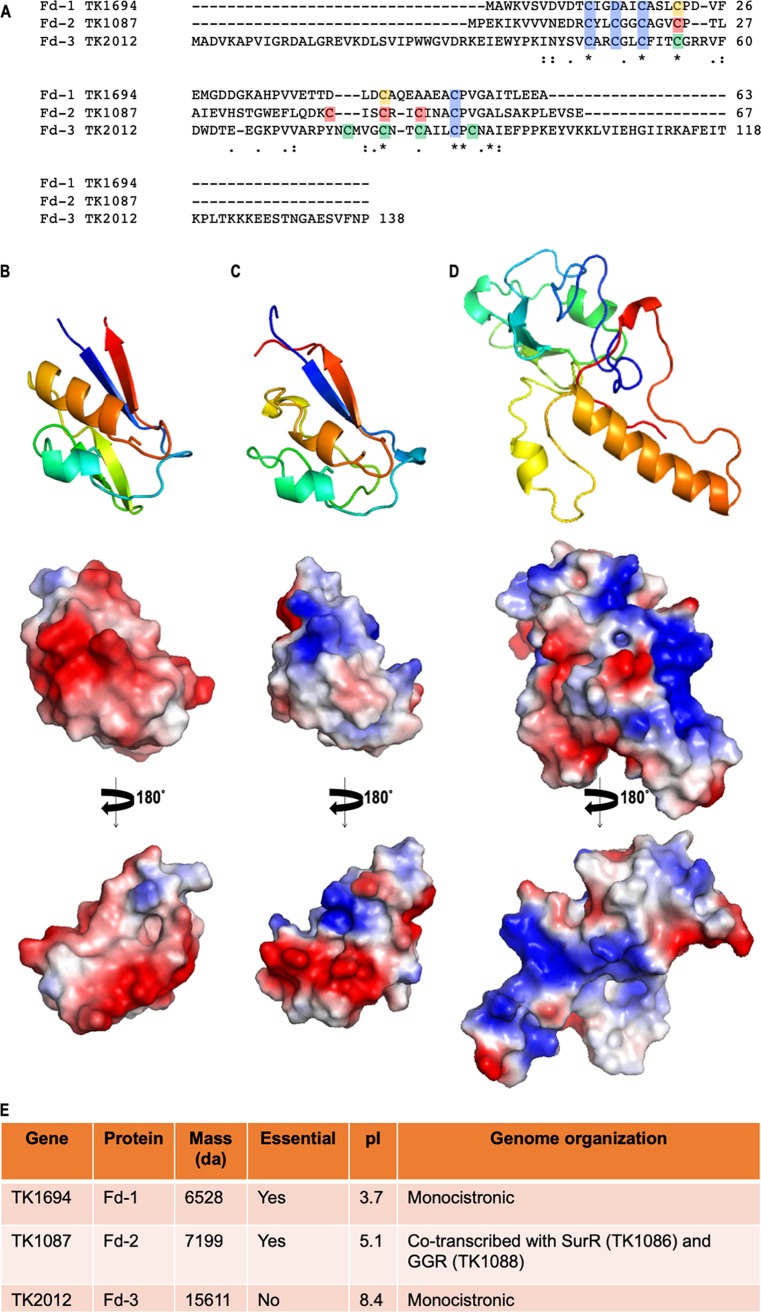
T. kodakarensis encodes three ferredoxins with dramatically different physical properties and expression patterns. (A) Sequence alignments of Fd-1, Fd-2, and Fd-3, highlighting residues likely involved in metal coordination (blue). (B, C, and D) Cartoon Phyre2 models of Fd-1 (B), Fd-2 (C), and Fd-3 (D) with electrostatic surface potentials shown below from two perspectives. (E) Summary of physical properties, essentiality, and genome organization of each Fd.

T. kodakarensis encodes three loci that were annotated (37) to encode putative Fds: TK1694 encodes Fd-1, TK1087 encodes Fd-2, and TK2012 encodes Fd-3 (Fig. 1). Unequivocal identification of Fds is nontrivial, with conservation of residues responsible for metal coordination often easily recognized in (CxxC)_2_ motifs, but little conservation of residues comprising the remainder of the protein (20, 21). Given the large number of redox-active, likely Fe-S-coordinating enzymes in T. kodakarensis, we cannot rule out that additional Fds are encoded within the genome.

Comparison of the primary sequences of the three annotated Fds reveals minimal consensus beyond the residues presumed responsible for metal binding (Fig. 1A). Fd-1 retains the greatest conservation to Fds that have been characterized in related species (48–50), and structural modeling (51) (Fig. 1B) predicts Fd-1 likely coordinates a 3Fe-4S center with three cysteines and a single aspartic acid residue (Fig. 1A, blue highlights). Like the well-studied Pyrococcus furiosus Fd (48), two additional cysteines are retained that may form a disulfide linkage to stabilize the overall fold of Fd-1 (Fig. 1A, orange highlights). Fd-2 and Fd-3 each retain four cysteines that could coordinate a single Fe-S center, as anticipated for Fd-1, but Fd-2 and Fd-3 additionally encode four or five cysteine residues, respectively, of unknown function. High-confidence-predicted structures (51) for Fd-2 (Fig. 1C) suggest that the additional four cysteines (Fig. 1A, rose highlights) are clustered close enough to permit disulfide-bond formation—with many possible cysteine-cysteine partnerships feasible—or coordinate an additional Fe-S center. Structural modeling (51) of Fd-3 (Fig. 1D) also returns high-confidence models, but none of these models brings together any combination of the nine cysteine residues (Fig. 1A, blue and green highlights) that would conceivably coordinate a single or multiple Fe-S centers.

Fd-1 and Fd-2 are similar in size (6.5 versus 7.2 kDa), both have acidic pIs (3.7 versus 5.1), and modeling predicts that these Fds share a similar overall fold. However, Fd-1 and Fd-2 have disparate electrostatic surface potentials, with nearly the entire surface of Fd-1 negatively charged, whereas the surface of Fd-2 has a mottled pattern of acidic and basic patches. Fd-3, in contrast, is approximately twice as large (15.6 kDa) and with an overall pI of 8.4 is distinctive from both Fd-1 and Fd-2 in size, predicted fold and shape, and overall charge densities. These dramatic differences in shape, charge, and size suggested that the Fds each present different surfaces for interactions with electron donors and acceptors. This implied that redox enzymes that utilize Fds either have Fd-specific interactions or present amorphous Fd-binding surfaces to permit promiscuous interactions with several Fds (52–54).

In addition to differences in size, shape, and charge that suggest distinct interactions for each Fd, the expression and cellular abundance of each Fd varies with the availability of different terminal electron acceptors and during different growth phases (44). Previous deep RNA sequencing (44) demonstrated that expression of Fd-1 is robust during exponential growth in both the presence and the absence of S^0^, the preferred terminal electron acceptor. Expression of Fd-2 and Fd-3, however, at the RNA level, is limited to just ∼1% of Fd-1 expression, and expression of Fd-2 and Fd-3 is dramatically and inversely impacted by the availability of S^0^. Fd-2 expression increases when S^0^ is available, whereas Fd-3 expression is not detectable at the RNA or protein level (see below) when S^0^ is present in the media. Fd-3 expression is maximized under S^0^-free conditions.

Fd-1 and Fd-3 appear to be monocistronic loci, but Fd-2 is the central gene in a three-gene operon. Fd-2, encoded by TK1087, is immediately downstream of the redox-responsive transcription factor SurR (46) and immediately upstream of the essential geranylgeranyl hydrogenase. Activity of the Methanosarcina geranylgeranyl hydrogenase in a heterologous host was dependent on the *Methanosarcina* ferredoxin encoded adjacently in the genome (43), suggesting that Fds and cognate Fd partners may be coexpressed under certain conditions.

### *In vivo* Fd partnerships are largely unique.

**FIG 2:**
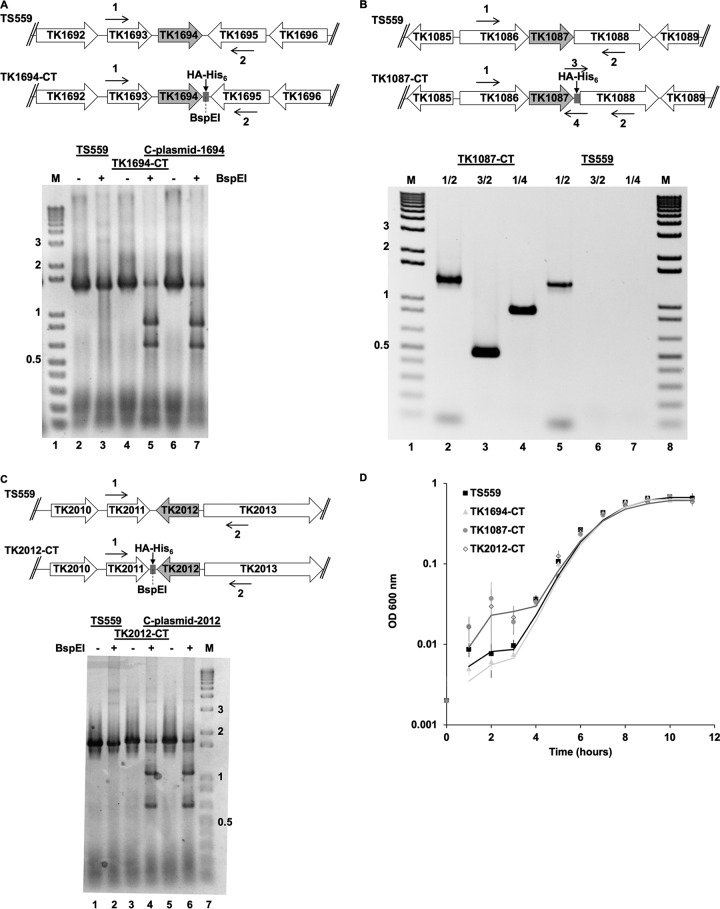
Loci encoding each Fd can be modified within T. kodakarensis to encode proteins with C-terminal extensions without compromising viability or growth rate. (A) Map of the TK1694 locus in the genomes of TS559 (top) and TK1694-CT (bottom), highlighting the approximate location of primer binding sites used to generate amplicons that were resolved either prior to (−) or following digestion with BspEI. Lane M in panels A, B, and C contains size markers in kbp. C-plasmid DNA for TK1694 was used as a control to confirm BspEI digestion of amplicon DNA. (B) Map of the TK1087 locus in the genomes of TS559 (top) and TK1087-CT (bottom), highlighting the approximate location of primer binding sites used to generate amplicons that were resolved in the gel below. Primers 1 and 2 are complementary to flanking sequences, while primers 3 and 4 are complementary to sequences within the 45-bp tag sequence at the C terminus of TK1087. Amplification with primer pairs 1 and 2 generates near-equivalent-size amplicons from genomic DNA from strains TS559 and TK1087-CT, while only DNA from TK1087-CT supports generation of amplicons with primer pairs 3 and 2 or 1 and 4. (C) Map of the TK2012 locus in the genomes of TS559 (top) and TK2012-CT (bottom), highlighting the approximate location of primer binding sites used to generate amplicons that were resolved either prior to (−) or following digestion with BspEI. C-plasmid DNA for TK2012 was used as a control to confirm BspEI digestion of amplicon DNA. (D) Strains with C-terminal extensions at individual Fd loci grow nearly identically to strain TS559 under standard laboratory conditions. The values plotted are the average of three assays each of triplicate cultures of each strain.

To examine the native partnerships and protein interactions made by each Fd under different physiological conditions, we generated three T. kodakarensis strains in which each Fd-encoding locus was extended by sequences that encode a C-terminal hemagglutinin (HA) epitope and His_6_ sequence (Table 1 and Fig. 2) (33). C-terminal extensions were predicted by modeling (51) not to interfere with folding or metal coordination, and C-terminal extensions ensure that any proteins resulting from alternative translation initiation positions retain the desired tags. These markerless genomic modifications importantly did not disrupt natural regulatory elements or result in polar repression of genes within operons. Insertion of the 45-bp tag-encoded sequences was confirmed by diagnostic PCR (Fig. 2A, B, and C), and all genomes retained in each strain were confirmed to be modified at the desired locus. Strains carrying Fd loci with extensions grew equivalently to the parental strain, TS559 (55), under all conditions tested, further demonstrating that genomic modifications resulting in Fd tagging did not interfere with Fd function *in vivo* (Fig. 2D).

**TABLE 1:** *T. kodakarensis* strains used or generated in this work

Strain	Genotype	Source or reference
TS559	Δ*pyrF* Δ*trpE*::*pyrF* ΔTK0664 ΔTK0149	55
TK1694-CT	TS559 with TK1694-HA-His_6_	This work
TK1087-CT	TS559 with TK1087-HA-His_6_	This work
TK2012-CT	TS559 with TK2012-HA-His_6_	This work
ΔTK2012 mutant	TS559 ΔTK2012	This work

**FIG 3:**
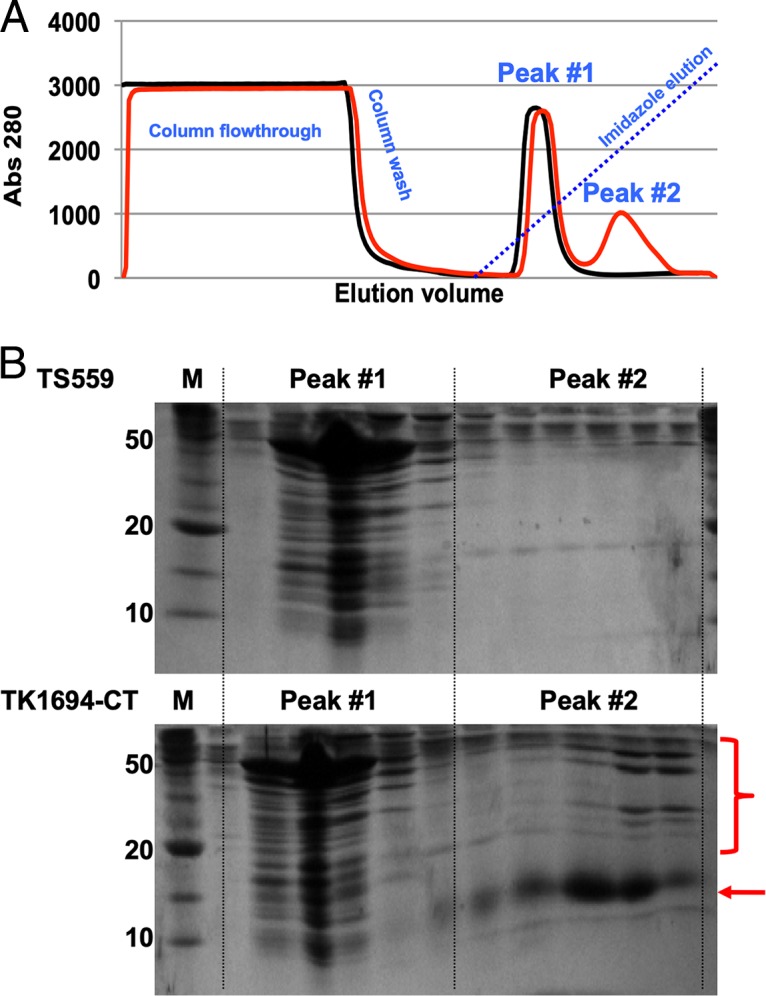
Fd proteins maintain stable interactions permitting copurification of Fd-binding partners. (A) Elution profiles of total clarified cell lysates of strains TS559 (black) and TK1694-CT (red) reveal an additional peak of absorbance at 280 nm (peak 2) that elutes at higher imidazole concentrations from lysates of TK1694-CT and which is not present in the parental strain, TS559. (B) SDS-PAGE of aliquots of fractions recovered from Ni^2+^-charged chelating chromatography from strains TS559 (top) and TK1694-CT (bottom) reveal near-identical protein patterns within peak 1, representing native T. kodakarensis proteins with mild affinity for the Ni^2+^-charged chelating matrix. Resolution of aliquots from peak 2 reveals minimal proteins in lysates derived from strain TS559, but obvious retention of Fd-1 (red arrow) and Fd-1-interacting proteins (red bracket) from lysates derived from strain TS1694-CT.

Rapid purification of Fds from cell lysates generated from mid-logarithmic cultures of each tagged-Fd strain was possible based on retention of tagged Fds on Ni^2+^-charged chelating columns under near-physiological conditions (33, 35, 56–58). Rapid cell lysis and subsequent chromatography resulted in retention of not only the tagged Fds, but also naturally occurring Fd-interacting proteins (Fig. 3). It is important to note that these interactions were formed under native conditions and at normal physiological concentrations of each Fd and redox factor. Identical growth, lysis, and chromatographic treatments of cell lysates derived from the parental strain TS559 demonstrated that >99% of total proteins were not retained on the matrix, and when imidazole gradients were applied to elute bound proteins, a single peak of proteins eluted at very low (∼10 mM) imidazole concentrations (Fig. 3). In contrast, when cell lysates from tagged-Fd strains were identically treated, in addition to the first low-imidazole-concentration-eluted protein peak, a second, broader peak of proteins eluted from the matrix at a higher imidazole concentration. Western blots using anti-HA antibodies unequivocally identified the tagged-Fd proteins in the second, higher-concentration imidazole-eluted protein peak (data not shown).

**FIG 4:**
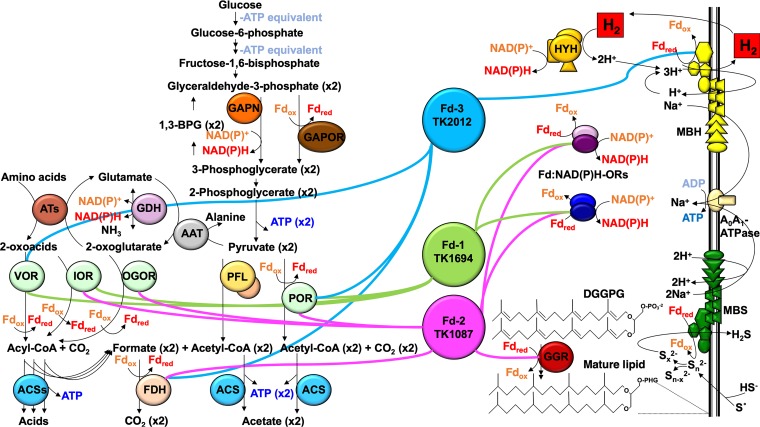
The T. kodakarensis Fd interactomes are distinct. The primary electron donors that reduced Fds are shown on the left within a model of glycolysis and amino acid fermentation. The primary electron acceptors that oxidize reduced Fds are shown to the right within partial representations of soluble lipid and NAD(P)H production pathways and membrane-bound respiratory and ATP-generating complexes. Each Fd is highlighted in the center of the panel, with interacting partners connected by solid, colored lines (Fd-1, green; Fd-2, pink; Fd-3, blue).

The proteins present in the second imidazole-dependent elution from the Ni^2+^-charged matrix were identified by MuDPIT (multidimensional protein identification techniques) analyses of pooled proteins (56). To control for low-abundance proteins that naturally elute from this matrix at higher imidazole concentrations even when not associated with tagged Fds, we also collected and analyzed with MuDPIT identically pooled fractions from lysates of TS559 that were chromatographed under the same conditions. By subtracting the proteins identified in TS559-derived fractions from those identified in fractions derived from strains carrying tagged-Fd loci, we generated Fd interaction networks for each Fd (Fig. 4 and Table 2).

**TABLE 2:** Ferredoxin interactomes identified in *T. kodakarensis*

TK gene no.	Protein function	S^0^ condition	No. of unique peptides	% of coverage	No. of normalized total spectra
Proteins copurified with TK1694 (Fd-1)					
TK1694	Ferredoxin 1	−S^0^/+S^0^	2	64	2,569
TK1980	2-Oxoisovalerate:ferredoxin oxidoreductase, α subunit	−S^0^/+S^0^	24	82	1,366
TK1981	2-Oxoisovalerate:ferredoxin oxidoreductase, β subunit	−S^0^/+S^0^	17	76	1,314
TK1978	2-Oxoisovalerate ferredoxin oxidoreductase, γ subunit	−S^0^/+S^0^	16	87	902
TK1325	Ferredoxin:NADH oxidoreductase, α subunit	−S^0^/+S^0^	25	66	319
TK1979	2-Oxoisovalerate ferredoxin oxidoreductase, δ subunit	−S^0^/+S^0^	8	88	290
TK0650	Rubrerythrin-related protein	−S^0^/+S^0^	15	67	157
TK1326	Ferredoxin:NADH oxidoreductase, β subunit	−S^0^/+S^0^	17	65	135
TK1684	Ferredoxin:NADP oxidoreductase, α subunit	−S^0^/+S^0^	23	65	125
TK1983	Pyruvate:ferredoxin oxidoreductase, α subunit	−S^0^/+S^0^	8	30	48
TK1685	Ferredoxin:NADH oxidoreductase, β subunit	−S^0^/+S^0^	8	39	40
TK1984	Pyruvate:ferredoxin oxidoreductase, β subunit	−S^0^/+S^0^	6	22	31
TK0537	Peroxiredoxin	+S^0^	2	10	19
TK1982	Pyruvate:ferredoxin oxidoreductase, δ subunit	−S^0^/+S^0^	2	33	13
TK0136	Indolepyruvate:ferredoxin oxidoreductase, α subunit	−S^0^/+S^0^	6	11	13
TK0135	Indolepyruvate:ferredoxin oxidoreductase, β subunit	−S^0^/+S^0^	1	4	2

Proteins copurified with TK1087 (Fd-2)					
TK1087	Ferredoxin 2	−S^0^/+S^0^	3	87	28
TK2076	Formate dehydrogenase, α subunit	−S^0^/+S^0^	31	54	887
TK1088	Geranylgeranyl hydrogenase	−S^0^/+S^0^	22	61	312
TK2077	Formate dehydrogenase, 4Fe-4S cluster-binding protein	−S^0^/+S^0^	10	67	185
TK2303	Thermosome, β subunit	−S^0^/+S^0^	21	54	182
TK0799	Type II DNA topoisomerase VI, subunit B	−S^0^/+S^0^	21	41	149
TK1325	Ferredoxin:NADH oxidoreductase, α subunit	+S^0^	16	51	133
TK1326	Ferredoxin:NADH oxidoreductase, β subunit	+S^0^	13	65	130
TK0798	Type II DNA topoisomerase VI, subunit A	−S^0^/+S^0^	12	30	119
TK2078	4Fe-4S cluster-binding protein associated with formate dehydrogenase	−S^0^/+S^0^	9	51	99
TK0893	Pyruvate formate-lyase activating enzyme-related protein	−S^0^/+S^0^	9	34	77
TK0678	Thermosome, α subunit	−S^0^/+S^0^	13	45	52
TK2075	4Fe-4S cluster-binding protein associated with formate dehydrogenase	−S^0^/+S^0^	4	38	44
TK1983	Pyruvate:ferredoxin oxidoreductase, α subunit	−S^0^/+S^0^	2	7	23
TK1984	Pyruvate:ferredoxin oxidoreductase, β subunit	−S^0^/+S^0^	3	15	21
TK1130	2-Oxoacid:ferredoxin oxidoreductases, α subunit	−S^0^/+S^0^	6	20	21
TK1056	Rubrerythrin-related protein	−S^0^/+S^0^	2	22	17
TK1123	Oxoacid:ferredoxin oxidoreductases, γ subunit	−S^0^/+S^0^	3	23	15
TK0136	Indolepyruvate:ferredoxin oxidoreductase, α subunit	−S^0^/+S^0^	3	7	10
TK2072	Cytosolic NiFe-hydrogenase, β subunit	−S^0^	2	7	7
TK2091	Membrane-bound hydrogenase, MbhL	−S^0^	3	10	6
TK1684	Ferredoxin:NADP oxidoreductase, α subunit	−S^0^	4	9	6
TK1055	Peroxiredoxin	−S^0^/+S^0^	2	16	5
TK2021	ATPase near Fe-transport operon	−S^0^/+S^0^	3	17	5
TK1685	Ferredoxin:NADH oxidoreductase, β subunit	−S^0^	2	10	4
TK1129	2-Oxoacid:ferredoxin oxidoreductases, β subunit	+S^0^	3	17	4

Proteins copurified with TK2012 (Fd-3)					
TK2012	Ferredoxin 3	−S^0^	21	77	77
TK2076	Formate dehydrogenase, α subunit	−S^0^	38	55	125
TK2091	Membrane-bound hydrogenase subunit, MbhL	−S^0^	5	10	27
TK1983	Pyruvate:ferredoxin oxidoreductase, α subunit	−S^0^	2	7	23
TK1085	Protein disulfide oxidoreductase	−S^0^	4	19	21
TK1596	V-type ATP synthase, subunit H	−S^0^	6	59	18
TK2090	Membrane-bound hydrogenase subunit, MbhK	−S^0^	6	47	15
TK1982	Pyruvate:ferredoxin oxidoreductase, δ subunit	−S^0^	2	33	13
TK1978	2-Oxoisovalerate ferredoxin oxidoreductase, γ subunit	−S^0^	4	25	12
TK1979	2-Oxoisovalerate ferredoxin oxidoreductase, δ subunit	−S^0^	3	47	11
TK2089	Membrane-bound hydrogenase subunit, MbhJ	−S^0^	4	24	9
TK0798	Type II DNA topoisomerase VI, subunit A	−S^0^	8	23	9
TK0537	Peroxiredoxin	−S^0^	3	19	8
TK1602	V-type ATP synthase, subunit A	−S^0^	4	9	7
TK2303	Thermosome, β subunit	−S^0^	5	11	6
TK2075	4Fe-4S cluster-binding protein associated with formate dehydrogenase	−S^0^	4	32	6
TK1603	V-type ATP synthase, subunit B	−S^0^	2	14	5
TK0525	Superoxide reductase	−S^0^	2	20	4
TK1604	V-type ATP synthase subunit D	−S^0^	2	9	3
TK2077	Formate dehydrogenase, 4Fe-4S cluster-binding protein	−S^0^	3	23	3
TK1984	Pyruvate:ferredoxin oxidoreductase, β subunit	−S^0^	2	12	2
TK0799	Type II DNA topoisomerase VI, subunit B	−S^0^	2	5	2
TK1981	2-Oxoisovalerate:ferredoxin oxidoreductase, β subunit	−S^0^	1	5	1
TK2088	Membrane-bound hydrogenase subunit, MbhI	−S^0^	1	8	1

Strains encoding tagged-Fd proteins were individually grown in medium lacking or containing S^0^ to identify the electron donors and acceptors that utilize each Fd under different physiological conditions. None of the Fds was identified in lysates derived from TS559, and only the unique Fd that was tagged in each strain was identified from lysates from each tagged-Fd strain. Fd-1 was identified only in lysates from strain TK1694-CT and was easily recovered from lysates derived from cultures grown in the absence and presence of S^0^. Fd-2 was identified only in lysates from strain TK1087-CT, and although not as abundant at the protein level as Fd-1, was easily detected by Western blots in late-eluting fractions from lysates derived from cultures grown in the absence and presence of S^0^. Fd-3 was identified only in lysates from strain TK2012-CT; however, the protein abundance of Fd-3 was only detectable by Western blotting and MuDPIT only from cultures grown in the absence of S^0^. Thus, the transcriptome sequencing (RNA-seq) (44) and crude protein levels are in agreement, with Fd-1 being the most abundant Fd under all conditions, Fd-2 present at modest levels −S^0^/+S^0^, and Fd-3 being detectable only in the absence of S^0^.

Stringent criteria were applied to identify bona fide Fd-interacting partners (see Materials and Methods). After eliminating proteins that were identified in lysates derived from TS559, the remaining candidate Fd interaction proteins were generally required to be identified by at least two peptides and to return a MASCOT score of >100. Although some valid partnerships were likely eliminated by our analyses, the results obtained, with minor exceptions, identified unique partnerships for each Fd. Single-peptide identification of proteins was permitted only in instances where multiple subunits of a known complex were coidentified with multiple peptides.

### Fd-1 is primarily involved in NAD(P)H production.

The protein interaction network (interactome) identified for Fd-1 suggests that while abundant, Fd-1 retains a minimal set of partnerships with central metabolism-based electron donors. Fd-1 copurifies only with pyruvate oxidoreductase (POR [TK1982, TK1983, and TK1984]), 2-oxoisovalarate-Fd oxidoreductase (VOR [TK1978, TK1979, TK1980, and TK1981]), and indolepyruvate-Fd oxidoreductase (IOR [TK0135 and TK0136]). Three, four, and two unique subunits of POR, VOR, and IOR, respectively, were recovered with Fd-1, giving confidence to these partnerships. In line with continued high-level expression of Fd-1 in the presence and absence of S^0^, the partnerships of Fd-1 largely did not change due to the availability of S^0^. The only additional Fd-1 partnership formed in the absence of S^0^ was with a peroxiredoxin (TK0537) shown to differentially oligomerize in response to oxidative and heat stress (59). Together with a rubrerythrin-related protein (TK0650), the Fd-1 interactome may assist in oxygen and peroxide detoxification strategies.

Electrons carried by Fd-1 do not appear to be broadly distributed, as high-confidence interactions could only be detected with two soluble Fd:NAD(P)H oxidoreductase complexes (42). Both subunits of both Fd:NAD(P)H oxidoreductases (TK1325-TK1326 and TK1684-TK1685) were returned as high-confidence partnerships. The thermo-instability of NAD(P)H (19), but requirement for NAD(P)H in many cellular transactions, likely requires a continuous supply of electrons through Fd-1 to meet cellular demands for NAD(P)H. Fd-1 did not retain any identifiable partnership with membrane-bound respiratory complexes.

### Fd-2 is primarily devoted to lipid maturation.

The redox interactome of Fd-2 is more extensive than that of Fd-1, despite the lower abundance of Fd-2 in clarified cell lysates. Like Fd-1, Fd-2 retains partnerships with several central metabolic enzymes, including POR and IOR, but Fd-2 does not partner with VOR. Fd-2 partnerships include high-confidence recovery of formate dehydrogenase (FDH) and maturation proteins for formate dehydrogenase (TK2075, TK2076, TK2077, and TK2078) and 2-oxoglutarate:Fd oxidoreductase (OGOR [TK1123, TK1129, and TK1130]). The recovery of several subunits of FDH and OGOR with Fd-2 suggests Fd-2 is the primary acceptor of electrons from FDH and the sole acceptor of electrons from OGOR.

The interactome of Fd-2 revealed several important electron acceptors, most uniquely geranylgeranyl hydrogenase (TK1088; alternatively termed geranylgeranyl reductase [GGR]), encoded immediately downstream of Fd-2 (TK1087). Lipid maturation is a major electron sink, and given the recovery of GGR only with Fd-2, coupled with cotranscription of Fd-2 and GGR within the same operon (44), Fd-2 appears to be the sole electron provider for GGR. Lower-confidence partnerships were returned with soluble Fd:NAD(P)H oxidoreductases, with interactions returned for Fd-2 with TK1325-TK1326 in the presence of S^0^, whereas TK1684-TK1685 were partnered with Fd-2 in the absence of S^0^. The abundance of the two soluble Fd:NAD(P)H oxidoreductases is known to be regulated in response to S^0^ availability (42).

Additional Fd-2 partnerships include a peroxiredoxin (TK1055) and a putative ATPase (TK2021) associated with an Fe transport operon (TK2018-TK2020). Whereas both Fd-1 and Fd-3 interact with a peroxiredoxin encoded by TK0537, Fd-2 partners with the peroxiredoxin encoded by TK1055 and the adjacently encoded rubrerythrin-related protein (TK1056) (59). Reducing equivalents are likely shuttled through Fds to oxygen and peroxide detoxification and stress responses.

The interactome of Fd-2 includes several unanticipated partners. Both subunits of the thermosome (TK2303 and TK0678) (60) were recovered with high confidence with Fd-2, as were the protein products of TK0798 and TK0799, encoding a type II DNA topoisomerase. Both thermosome subunits and both topoisomerase subunits were also recovered with Fd-3 (see below). Finally, fractions containing Fd-2 from cultures grown in the absence of S^0^ contain detectable levels of one subunit of the cytoplasmic soluble H_2_-reducing hydrogenase (TK2072), a pyruvate formate lyase-activating enzyme (TK0893), and a subunit of the hydrogen-evolving membrane-bound hydrogenase (MBH [TK2091]). Only a single subunit of each hydrogenase was recovered that met our stringent criteria for interaction. No additional subunits of either complex could be identified in Fd-2-containing fractions even when criteria were lowered to include single-peptide identifiable proteins, and TK2072 and TK2091 were only recovered under S^0^-free conditions, wherein expression of both hydrogenases is known to increase (9, 44). Direct interaction of any Fd with the cytosolic hydrogenase was not predicted, and although Fd interaction with MBH was anticipated, the catalytic subunit of MBH was not recovered with Fd-2, but was returned in high confidence with Fd-3 (see below).

### Fd-3 is primarily devoted to H_2_ evolution.

Fd-3 expression could only be detected at the RNA level in the absence of S^0^ (44), and we were also unable to detect Fd-3 expression at the protein level when S^0^ was present in the culture medium. In S^0^-free cultures, Fd-3 expression was confirmed via Western blotting, and Fd-3 and interacting proteins could be identified by MuDPIT. The low abundance of Fd-3 likely resulted in low-abundance recovery of interacting partners, but high-confidence protein identifications were nonetheless possible. Fd-3 maintains interactions with FDH (TK2075, TK0276, and TK2077), but recovery of FDH subunits with Fd-2 was much greater than with Fd-3. As was seen for Fd-1, very-high-confidence protein identifications were made for several subunits of POR (TK1982, TK1983, and TK1984) in Fd-3-containing lysates. Fd-3 maintains identifiable interactions with VOR (TK1978, TK1979, and TK1981), but to the exclusion of OGOR and IOR. Thus, electrons derived from fermentation of amino acids are parsed through each Fd, with OGOR specific to Fd-2, VOR partnering with Fd-1 and Fd-3, IOR reducing either Fd-1 or Fd-2, and POR employing all three Fds for electron transfer.

Electrons carried by Fd-3 appear to be destined primarily for the MBH complex and H_2_ evolution coupled to generation of an electrochemical gradient for ATP production (10, 14, 40). Fd-3 returns high-confidence partnerships with at least four MBH subunits (TK2091, TK2090, TK2089, and TK2088), importantly including the known active-site-containing cytoplasmic subunit MbhL (TK2090). Although a direct interaction with the Na^+^-dependent V-type ATPase was not predicted, Fd-3 uniquely copurifies not only with MBH but also with at least four subunits of the ATPase (TK1596, TK1602, TK1603, and TK1604). Copurification raises the possibility that MBH and the ATPase are physically associated *in vivo*. Fd-3 also appears to donate electrons to combat oxidative stress, via partnerships with peroxiredoxin (TK0537), superoxide reductase (TK0525), and thioredoxin/protein-disulfide oxidoreductase (TK1085).

### Fd partnerships with MBS and GAPOR were not recovered.

The membrane-bound sulfane reductase (MBS; previously known as the membrane-bound oxidoreductase, MBX [TK1214–1226]) (15) and the glyceraldehyde-3-phosphate:Fd oxidoreductase (GAPOR [TK2163]) (36, 39, 61) were the only major electron acceptor and donor to not be returned with a stable Fd partnership. Purified P. furiosus MBS can utilize electrons from Fd *in vitro* to reduce polysulfides while generating an electrochemical gradient for ATP production (15). MBS expression is highest in the presence of S^0^ (9, 44), and thus, Fd-1 and/or Fd-2 was predicted to serve as an electron carrier to MBS. The failure to return an Fd partnership with MBS suggests that (i) the Fd-MBS interaction is weak or transient, (ii) an additional Fd is encoded in T. kodakarensis, or (iii) MBS subunits are difficult to solubilize and recover from clarified cell lysates. It is also formally possible that in T. kodakarensis, a direct Fd-MBS interaction is not present, but rather that electrons are donated to MBS through some intermediate electron carrier. Based on our analyses, the only likely electron donor for MBS would be NAD(P)H. Although energetically possible based on midpoint electrical potentials of −320 mV for NAD(P)H and just ∼−270 mV for polysulfides (5), it remains to be tested if such an electron transfer would be sufficiently exergonic to drive MBS to expel protons to generate an electrochemical gradient.

GAPOR is nonessential under gluconeogenic conditions, but required for glycolytic growth (61). Our culture conditions primarily result in gluconeogenic growth (62). We expect the failure to recover a GAPOR-Fd interaction results from little to no expression of GAPOR under our experimental conditions (36).

### Fd-1 and Fd-2 are essential, but Fd-3 is dispensable, under S^0^-containing conditions.

The distinct Fd interactomes suggested that each Fd plays an essential role in electron flux *in vivo* and that each Fd might therefore be required for viability. Despite extensive efforts, we were unable to generate T. kodakarensis strains wherein sequences encoding Fd-1 (TK1694) or Fd-2 (TK1087) were deleted from the genome. Efforts to generate such deletion strains failed under all physiological conditions. The markerless procedures employed (33) were carefully designed in the case of Fd-2 to ensure that expression of the downstream locus (TK1088, encoding GGR) was not affected by deletion of TK1087 coding sequences. The essentiality of Fd-1 is most easily explained by its cellular abundance and the need for continual electron flux through NAD(P)H:Fd oxidoreductases to replenish NAD(P)H pools. The essentiality of Fd-2 is perhaps most easily explained by the unique partnerships with both OGOR and GGR and the continual requirement for reducing equivalents for maturation of archaeal isoprenoid-based lipids.

**FIG 5:**
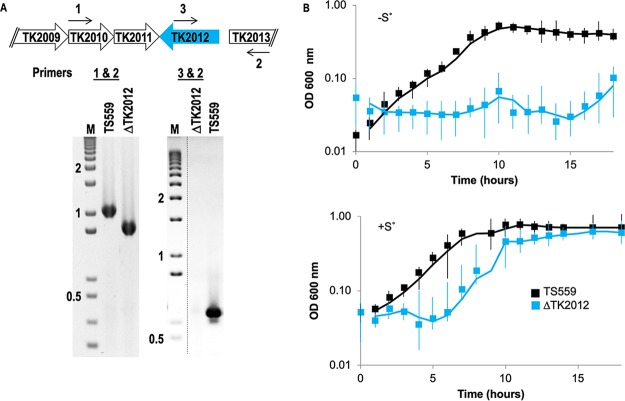
TK2012, encoding Fd-3, is nonessential in the presence of S^0^. (A) Map of the TK2012 locus in the genome of TS559 highlighting the approximate location of primer binding sites used to generate amplicons that were resolved in the gel below. Primers 1 and 2 are complementary to TK2012 flanking sequences, while primer 3 is complementary to sequences within TK2012. Amplification with primer pairs 1 and 2 generates a shorter amplicon from genomic DNA from the ΔTK2012 strain than DNA from strain TS559, reflecting the loss of TK2012 coding sequences. Only DNA from TS559 supports generation of an amplicon with primer pair 1 and 3. Lane M contains size markers in kbp. (B) Growth of strains TS559 and ΔTK2012 in the presence of S^0^ (lower panel) is robust, whereas the ΔTK2012 strain fails to achieve significant densities in the absence of S^0^ (top panel).

The expression data at the RNA and protein levels suggested that Fd-3 might be dispensable under S^0^-containing conditions, given that neither Fd-3 transcripts nor protein can normally be detected under such conditions. As predicted, we were able to markerlessly delete the sequences encoding Fd-3 (TK2012) from the TS559 genome under conditions where S^0^ was retained in all media. Deletion of TK2012 was confirmed by diagnostic PCRs (Fig. 5A). All efforts to generate a strain deleted of Fd-3 in the absence of S^0^ were unsuccessful.

Given that the most unique partnerships of Fd-3 were with MBH, which itself is dispensable under S^0^-containing conditions but required under S^0^-free conditions (10), we anticipated that if Fd-3 was the exclusive or dominant electron donor to MBH, strains deleted for Fd-3 would likewise fail to grow under S^0^-free conditions. This hypothesis was validated by following culture growth of strains TS559 and ΔTK2012 under optimized conditions in the presence and absence of S^0^ (Fig. 5B). Minimal effects of Fd-3 deletion were observed under S^0^-containing conditions, whereas essentially no growth was observed for strain ΔTK2012 when S^0^ was removed from the medium. The inability of the ΔTK2012 strain to grow in the absence of S^0^ is fully supportive of MBH accepting electrons from only a single Fd—in this case, Fd-3—and the nonoverlapping and distinct physiological roles of each Fd in T. kodakarensis.

## DISCUSSION

Redox reactions and electron flux are critical to all metabolisms. Fds—an ancient class of Fe-S-containing small proteins—are the most widely distributed and prevalent proteinaceous electron carriers (20, 21). Almost all Bacteria, Archaea, and Eukarya encode multiple Fds, implying that the redox balance of most cells is coordinated by the combined and intertwined activities of each Fd. With rare exception (22), the *in vivo* partnerships, expression levels, and electrical potentials that dictate electron flow through Fd isoforms are not known. As both a greater understanding of diverse microbial metabolisms emerges—and bioengineering efforts in many species increase—so does interest in understanding the maximum carrying capacity of Fd isoforms, their dominant electron donors and acceptors, their dispensability or essentiality, and their ability to be differentially regulated under unique growth conditions.

Here we take advantage of the flexible metabolism and facile genetic system (33–35) of the model hyperthermophilic archaeon T. kodakarensis to determine the physiological roles of three Fds encoded in the genome. Deep RNA-seq revealed that each Fd is differently expressed at the RNA level (44), and Western blotting demonstrated differences in Fd isoform abundance during exponential growth. The differences in Fd isoform size, shape, overall charge, distributed electrostatic surface potentials, and likely electrical potential suggested that each Fd would make specific contacts with select electron donors and acceptors. We demonstrate—through identification of native, *in vivo*-derived natural protein-protein partnerships—that each Fd isoform largely makes unique contributions to electron flux and physiology of T. kodakarensis under different conditions (Fig. 4). Our results are consistent with each Fd functioning as a stand-alone electron carrier, rather than as a stable component of a redox assembly.

Fd-1 is highly abundant and is primarily involved in transferring electrons from glycolysis and amino acid fermentation to oxidoreductases that reduce NAD(P)+. Fd-2 is modestly abundant in S^0^-free medium, and expression is increased when S^0^ is available. In contrast to Fd-1, Fd-2 is reduced by FDH, IOR, and OGOR. Reduced Fd-2 primarily interacts with GGR to provide reducing equivalents for lipid maturation, although some interactions could be detected that could result in NAD(P)H production. The unique roles of Fd-1 and Fd-2 in NAD(P)H production and lipid maturation/FDH function, respectively, are supportive of our findings that the loci encoding these Fd isoforms cannot be deleted from the T. kodakarensis genome. Although we find interactions between Fd-2 and NAD(P)H:Fd oxidoreductases, the electron flux through Fd-2 is unlikely to support sufficient NAD(P)H production to support growth without electron flux through Fd-1. Likewise, only Fd-2 demonstrates interactions with GGR, and the essentiality of TK1087 implies that neither Fd-1 nor Fd-3 can substitute for Fd-2 in supplying electrons for lipid reduction. Given that Fd-2 is present at only a small percentage of the levels of Fd-1, the failure of Fd-1 to support growth in the absence of Fd-2 suggests that the Fd-2–GGR partnership is specific and cannot be rescued by even promiscuous Fd-1–GGR interactions.

Fd-3 also retains a unique interactome, distinct from Fd-1 and Fd-2. Fd-3 appears to be reduced by POR, VOR, and FDH, and unlike Fd-1 and Fd-2, is dispensable when S^0^ is present in the environment. Supportive of the detected interactions uniquely between Fd-3 and MBH, deletion of Fd-3 results in T. kodakarensis strains that cannot grow in the absence of S^0^. Our results imply that electron flux to MBH is dependent on Fd-3 and that Fd-1 and Fd-2 are not capable of substituting for Fd-3 to support growth in the absence of S^0^. The use of T. kodakarensis and related species as hydrogen-evolving platforms (9–11, 16, 18, 38, 63–67) must take notice of this finding, as flux through MBH may be limited by flux through Fd-3.

The metabolism of T. kodakarensis and many closely related species has identified a large number of redox-active enzymes, and with only two notable exceptions, we were able to identify the Fd isoform(s) that provides electron-carrying capacity to these reactions. The reduction of polysulfides, formed spontaneously at high temperatures by reaction of S^0^ and H_2_S, provides a route of electron disposal, and when catalyzed by the MBS complex, can generate an electrochemical gradient useful for ATP production (15). Our identification of Fd-3–MBH partnerships suggests that MBH and MBS were at least partially solubilized during formation of clarified cell lysates. Additional studies will be required to determine if T. kodakarensis MBS directly accepts electrons from Fd, as demonstrated for P. furiosus MBS *in vitro*. Deletion of MBS from either T. kodakarensis or P. furiosus is nonphenotypic when S^0^ is present, whereas deletion significantly impairs but does not block growth in the absence of S (10, 15).

The relatively long-lived partnerships that were selectively retained for each Fd challenge the notion that many redox enzymes can utilize any Fd isoform available to them. Instead, our results can be extrapolated to suggest that most Fd isoforms—in most species—are employed to shuttle electrons between distinct and unique sets of electron donors and acceptors. The added level of regulation on overall electron flux that can be afforded by differentially expressing and controlling the levels of each Fd *in vivo*, combined with unique partnerships between some redox enzymes and Fds, likely contributes to the fitness of many species under changing and challenging environmental conditions.

Challenges remain in determining the importance of specific Fd isoforms in the metabolism of most cells. Unequivocal identification of Fds is a major hurdle, as conservation of sequence or structure is not common outside the residues responsible for coordinating the metal clusters. Determination of electric potentials remains problematic, and *in silico* predictions of Fd partnerships based on Fd primary sequence are required. Finally, bioengineering strategies toward production of high-value products in various microbial and model hosts must account for the limited flux through and distinct partnerships of Fd isoforms.

## MATERIALS AND METHODS

### Microbial strains and medium formulations.

T. kodakarensis strains were maintained under strict anaerobic conditions at 85˚C in artificial seawater-based medium (ASW) supplemented with a vitamin mixture and trace minerals, as previously described (33). The medium was solidified by addition of 1% Gelzan. Minimal medium contained amino acids as carbon sources, while rich medium contained 5 g/liter yeast extract and 5 g/liter tryptone. When present, 2 g/liter S^0^ was added to liquid cultures, but S^0^ was replaced by polysulfides in solid medium formulations (33). When S^0^ or polysulfides were not included in the media, 5 g/liter pyruvate was supplied. The culture headspaces were 95% N_2_–5% H_2_ at 1 atm at the time of inoculation. When necessary, agmatine was added to 1 mM and 6-methylpurine was added to 100 µM. All strains generated here originate from the parental strain, TS559 (33, 55). Microbial growth was followed by changes in optical density at 600 nm (OD_600_).

### Construction of tagged- and deleted-Fd strains.

Strain construction followed established procedures (33). Briefly, transformations of TS559 with nonreplicating plasmids—C-plasmids for C-terminal tags or B-plasmids for target deletion—permitted selection of individual strains wherein the loci encoding each ferredoxin (TK1694, TK1087, and TK2012) were targeted via homologous sequences for integration of the nonreplicating plasmids. Intermediate strains containing the entire plasmid sequence integrated at the target locus were selected on solid medium lacking agmatine and confirmed by diagnostic PCRs using purified genomic DNA. Confirmed intermediate strains were grown overnight in rich medium containing agmatine and then plated on solid amino-acid-based medium containing agmatine and 6-methylpurine. Excision of the plasmid sequences and simultaneous addition of 45 bp to the C terminus or deletion of each target locus were confirmed via diagnostic PCRs from genomic DNAs and sequencing of amplicons containing the target locus. The inserted 45-bp sequences introduce a new BspEI restriction site, permitting identification of tagged strains via comparison of BspEI digestion patterns of amplicons containing the target locus generated from genomic DNAs from TS559 and potential tagged strains. Deletion of the target locus results in shorter amplicons when primers flanking the target locus are employed in diagnostic PCRs. The exact endpoints of the deletion of TK2012 were confirmed by sequencing of amplicons generated from genomic DNA isolated from the ΔTK2012 strain.

### Purification and identification of the Fd interactomes.

One-liter cultures of strains TS559, TK1694-CT, TK1087-CT, and TK2012-CT were grown to early exponential phase (OD of ∼0.2 to 0.4) in ASW-yeast extract-tryptone-agmatine medium containing either S^0^ or pyruvate. Cultures were rapidly chilled to 4˚C, and all procedures were carried out at 4˚C. Biomass was harvested via centrifugation (∼20,000 × *g*, 20 min) and lysed via repeated sonication in 3 ml/g of biomass in a mixture of 25 mM Tris-HCl (pH 8.0), 500 mM NaCl, and 10% glycerol (buffer A). Cellular debris was cleared via centrifugation (∼20,000 × *g*, 20 min), and the clarified lysates were applied to 5 ml Ni^2+^-charged chelating columns equilibrated with buffer A. Columns were exhaustively washed with buffer A until no additional proteins eluted, and then bound proteins were eluted with a linear imidazole gradient to 100% buffer B (25 ml Tris-HCl [pH 8.0], 100 mM NaCl, 10% glycerol, 200 mM imidazole). Fractions were resolved via SDS-PAGE, transferred to polyvinylidene difluoride (PVDF), and probed with anti-HA antibodies (Covance Research, but now marketed by BioLegend; Covance no. MMS-101R; BioLegend 901513 [both stem from clone 16B12]) as described previously (33, 68). Fractions containing tagged Fds were pooled, as were equivalent fractions from identical procedures on strain TS559, and the proteins within were precipitated by addition of trichloroacetic acid to 15% (wt/vol). Proteins were quantified via Bradford assays (69) and submitted to the Ohio State Proteomics facility for MuDPIT analyses as described previously (56, 58). Proteins identified by at least two unique peptides in samples from tagged strains that were not identified in identical fractions from strain TS559 are listed in Table 1, ranked by total normalized spectra. The total number of unique peptides identified, whether proteins were recovered in the presence or absence of S^0^ in the media, and the percentage of the total protein sequence that was recovered in all unique peptides identified are also reported. Protein identifications based on a single unique peptide were only included when additional proteins within a known protein complex were also identified in the same sample.
